# A modified method for purification of *Eimeria tenella* sporozoites

**DOI:** 10.1007/s00436-020-06602-w

**Published:** 2020-01-16

**Authors:** Zaida Rentería-Solís, Runhui Zhang, Shahinaz Taha, Arwid Daugschies

**Affiliations:** 1 Institute for Parasitology, Faculty of Veterinary Medicine, Centre for Infectious Diseases, University of Leipzig, An den Tierkliniken 35, 04103 Leipzig, Germany; 2 Albrecht-Daniel-Thaer-Institute, An den Tierkliniken 29, 04103 Leipzig, Germany

**Keywords:** *Eimeria*, Coccidiosis, Chicken, Sporozoite, Purification, Excystation

## Abstract

Coccidiosis is an economically important gastrointestinal disease in domestic fowl. *Eimeria* species are the causative agents of avian coccidiosis. Current challenges in management and prevention of eimeriosis enhance the need for research in this field. Sporozoite purification is a necessary step for *Eimeria* spp. in vitro infection models. Current alternatives such as DE-52 anion exchange chromatography and Percoll gradient require time and resources. We present a modified protocol consisting on vacuum filtration of sporozoites using a disposable 5-μL filter. Yield percentages were similar to those reported for Percoll gradient purification. By reducing time and efforts during sporozoite purification, it could be possible to increase resources in other areas of *Eimeria* studies.

## Introduction

Coccidiosis is one of the most important diseases of the poultry industry. It can cause economic losses of over 800 million US dollars annually worldwide (Shirley et al. 2007). *Eimeria* spp. are the cause of coccidiosis in domestic fowl. These parasites cause gastrointestinal problems characterised by diarrhoea, weight loss, reduced egg production and in some cases death. In chickens, seven *Eimeria* spp. are commonly found in the field. They differentiate themselves in oocyst morphology, virulence and area of infection in the digestive system. Additionally, infection with *Eimeria* spp. can promote opportunistic infections by other pathogens like bacteria (Collier et al. 2008). *Eimeria* oocysts are highly tolerant to the environment, which makes control measures difficult. Additionally, the common prophylactic use of anticoccidial feed additives has led to wide spread anticoccidial resistance (Stephan et al. 1997). *Eimeria tenella* is one of the most frequent and pathogenic *Eimeria* spp. in domestic fowl (Blake et al. 2015). It can cause haemorrhagic caecal disease with fatal outcome in some cases. Given its importance, *E. tenella* is commonly studied and used as a model for chicken coccidiosis (Blake et al. 2015, Hiob et al. 2017, Thabet et al. 2017, Zhang et al. 2018). Nevertheless, there is still a large need for more research within this field. A valuable tool regularly used as an alternative to in vivo model is in vitro infection assay (Dimier-Poisson et al. 2004, Thabet et al. 2017). Purification of *Eimeria* sporozoites is an important step before cell culture infection. There are currently some purification methods available (Schmatz et al. 1984, Dulski and Turner 1988, Zhang et al. 2015). Purification of *E. tenella* sporozoites by DE-52 anion exchange chromatography is a method regularly used during in vitro studies (Schmatz et al. 1984). Nevertheless, this technique involves time and additional materials. Similarly, the use of Percoll gradients also requires time for gradient preparation (Dulski and Turner 1988). In this study, we present a simple alternative for *E. tenella* sporozoite purification.

## Material and methods

*Eimeria tenella* Houghton strain (kindly provided by Prof. Dr. D. P. Blake, Royal Veterinary College, University of London, UK) was passaged in healthy 10-day old chickens. Oocysts were purified from the faeces of the chickens following a modified protocol (Eckert et al. 1995). Briefly, faecal samples were collected and transferred to a 5-L plastic bucket. Thereafter, 2 volumes of tap water were added. Then, samples were homogenised with a hand blender (Braun, Frankfurt, Germany) until the mixture was homogeneous. The homogenate was filtered through a 250-μm-pore-size sieve and the filtrate transferred to a 2-L cylinder and sedimented overnight. Afterwards, the supernatant was discarded, and the sediment was resuspended in saturated saline solution. Followed by centrifugation of the resuspension at 1300×*g* for 10 min. Afterwards, suspended oocysts were collected and washed with tap water by centrifugation at 1300×*g* for 10 min. Finally, purified oocysts were collected and incubated for sporulation in 4% potassium dichromate solution at room temperature for 48 h. Sporulated oocysts were stored at 4 °C until further use.

The entire protocol was performed in triplicate with a total amount of 1.5 × 10^5^ oocysts per replicate. Before excystation, sporulated oocysts were cleaned from the potassium dichromate through centrifugation. Briefly, oocysts were centrifuged at 1300×*g* for 10 min at room temperature (RT). Afterwards, the supernatant was discarded and the pellet was resuspended in sterile PBS (pH 7.0) and centrifuged again at 1300×g for 10 min at RT. This centrifugation step was repeated twice or until the supernatant was clear. Oocysts’ surface sterilisation was performed as follows: after the last centrifugation of the previous step, the pellet was resuspended in 12% sodium hypochlorite (Carl-Roth, Karlsruhe, Germany) and incubated in a tube mixer at room temperature for 10 min. Immediately afterwards, the oocysts were centrifuged at 2500×*g* for 3 min. After centrifugation, the white cloudy layer formed at the top of the supernatant was transferred to clean 50-mL tube and resuspended in sterile PBS (pH 7.0). Oocysts were cleaned from sodium hypochlorite through vacuum filtration using a sterile1-μm Pluristrainer® filter (Pluriselect, Leipzig, Germany) mounted on a sterile connector ring (Pluriselect, Leipzig, Germany). After filtration, the filter was washed 3 times with sterile PBS (pH 7.0) in order to recover the oocysts. The filtration step was repeated once more. After the second filtration, recovery of oocysts was done using sterile PBS with pH 7.6–8.0. Alternatively, the oocysts can also be cleaned from the sodium hypochlorite by centrifugation at 1300*g* for 10 min, followed by 3 wash cycles with PBS at 1300*g* for 10 min. The recovered oocysts were immediately transferred to a 15-mL falcon tube with 0.5-mm sterilised glass beads (Carl Roth, Karlsruhe, Germany). The glass beads filled the tube up to the 0.5- to 1-mL mark. Once added to the tube with glass beads, the oocyst suspension was filled with sterile PBS (pH 7.6–8.0) up to 4 mL. Release of the oocysts’ wall was performed by vortexing the tubes for 3 cycles of 20 s. After each 20 s cycle, the sporocyst-oocyst ratio was examined using a light microscope. If necessary, an additional 20-s cycle was performed in order to maximise the number of released sporocysts. Afterwards, the supernatant with released sporocysts was transferred to a 15-mL falcon tube. The glass beads were washed 2–3 times with 3 mL of sterile PBS (pH 7.6–8.0) to collect remaining sporocysts. Later on, sporocysts were centrifuged at 2500×*g* for 10 min at RT. The supernatant was discarded and the pelleted sporocysts were enzymatically excysted. Briefly, sporocysts were incubated with 0.25% trypsin (Biochrom AG, Berlin, Germany) and 4% sodium taurocholic acid (Sigma, Taufkirchen, Germany) in sterile PBS (pH 7.6–8.0) at 41 °C for 60 to 90 min. Monitoring of excystation rate was performed every 30 min with light microscopy. After incubation, free sporozoites were transferred to 50 mL of 1% glucose (Carl Roth GmbH, Karsruhe, Germany) in sterile PBS (pH 7.0) previously warmed to 41 °C. Immediately afterwards, sporozoites were purified by vacuum filtration, using a 5-μm Pluristrainer® filter (Pluriselect, Leipzig, Germany) mounted on a sterile connector ring (Pluriselect, Leipzig, Germany). To reduce the amount of sporocyst residue, sporozoites can also be filtered by gravity. For this option, the sterile connector ring is superfluous. After filtration, sporozoites are washed from the glucose solution by centrifugation at 3200×*g* for 10 min at RT. Right thereafter, the supernatant is carefully removed and the sporozoites are resuspended in the appropriate infection medium. Finally, sporozoites are counted under a light microscope using a Neubauer chamber (depth 0.100 mm, Paul Marienfeld GmbH, Lauda-Königshofen, Germany).

Sporozoite viability after purification was assessed according to Thabet et al. (2015). Briefly, Madin-Darby Bovine Kidney Cells (MDBK) were seeded in 24-well plates with Dulbecco’s Modified Eagle’s Medium (DMEM) with 5% foetal bovine serum (FBS), 100 IU penicillin, 100 μg/mL streptomycin and 2.5 μg/mL amphotericin B. Cells were incubated at 37 °C and 5% CO_2_ until they reached 80 to 90% confluence. Cells were infected with freshly purified *E. tenella* sporozoites (5 × 10^4^/well). The negative control group (NC) consisted of uninfected MDBK cells. All groups were performed in triplicate. Following infection, all groups of cell cultures were incubated at 41 °C and 5% CO_2_ for 24 h. After 24 h, cells were washed 3 times with sterile PBS (pH 7.0) and fresh DMEM was added. After incubation at 41 °C for 96 h DNA was extracted from the cells using the DNeasy Blood & Tissue kit (Qiagen, Hilden, Germany) following manufacturer’s instructions. Quantification of *E. tenella* genomic copies was performed in triplicate using a real-time PCR assay. Additionally a non-template control (NTC) consisting of nuclease-free water was added in triplicate to the assay. The primers ETF 5′-TGGAGGGGATTATGAGAGGA-3′ and ETR 5′-CAAGCAGCATGTAACGGAGA-3′ were used to amplify a 147-bp fragment of the *E. tenella* internal transcribed spacer 1 (ITS-1) gene using a SYBR Green-based assay (Thermo Scientific, Darmstadt, Germany) according to Kawahara et al. (2008). Finally, the number of gene copies was calculated from the qPCR data by using a standard curve prepared from a serial dilution of cloned ITS-1 gene fragment according to Thabet et al. (2015).

## Results and discussion

Sporozoites recovered using the described protocol were clean and motile. After oocysts sterilisation with 12% sodium hypochlorite, yield efficacy was calculated for every further step of the procedure (Table 1). Overall, a yield of 35.69 ± 8.93% was recorded. Parasite loss was highest after grinding of oocysts with a yield mean of 55.55 ± 9.17% (Table 1). A low loss of sporozoites was calculated for the purification step with a mean value of 3.33%. Sporozoites collected by the current procedure were viable as assessed by successful in vitro cell invasion and replication. An initial number of 1.21 ± 0.39 × 10^5^ gene copies of the selected *E. tenella* gene fragment was determined by qPCR in 3 μL of total DNA after 96 h of incubation of MDBK following infection with 4 × 10^4^ sporozoites.

**Table 1:** Parasite recovery and yield efficacy

Step	Recovered parasitic stage	Quantity of parasites after each step^a^	Yield (%)^a,b^
Cleaning of oocysts with 1-μm filter^c^	Oocysts	1.19 ± 0.13 × 10^5^	79.44 ± 9.17
Oocysts mechanical grind with glass beads	Sporocysts	3.33 ± 0.55 × 10^5^	55.55 ± 9.17
Excystation	Sporozoites	4.46 ± 0.9 × 10^5^	39.02 ± 7.50
Sporozoites purification with 5-μm filter	Sporozoites	4.28 ± 1.07 × 10^5^	35.69 ± 8.93

^a^Quantities and yields reported are the means ± standard deviations of triplicate

^b^Yield percentages calculated from an initial amount of 1.5 × 10^5^ oocysts

^c^Yield percentages of earlier steps during oocysts cleaning were not calculated

We present a modified protocol for *E. tenella* sporozoite purification. An earlier version of this protocol has been successfully implemented in in vitro studies (Zhang et al. unpublished data, Rentería-Solís et al. unpublished data). Purification of *E. tenella* sporozoites can also successfully be performed by other protocols such as DE-52 anion exchange chromatography (Schmarz et al. 1984), Percoll gradients (Dulski an Turner 1988, Thabet et al. 2015) or with a 1400-mesh filter (Zhang et al. 2015). The protocol described in this report uses a plastic disposable 5-μm filter that perfectly fits to a 50-mL falcon tube. In this study, the Pluristrainer® model from Pluriselect (Leipzig, Germany) was used; however, similar products by other manufacturers exist in the market and are probably equally suited. By using a disposable 5-μL filter, there is no need of applying additional purification steps. This reduces the costs and time spent on sporozoite purification significantly thus saving resources in laboratory approaches.

Dulski and Turner (1988) reported a total yield of 39% of sporozoites using Percoll gradient for purification. Similar efficacy was obtained with the method presented in this study with 35.69 ± 8.93% of sporozoites recovered. Interestingly, Schmatz et al. (1984) reported a recovery of between 94 and 100% sporozoites after DE-52 anion exchange chromatography. However, the authors do not specify if the recovery percentage is calculated based on the initial amount of oocysts or in relation to the number of sporozoites collected during the previous step of purification. In our study, only 55 ± 9.17% of sporocysts were recovered after grinding of the oocysts’ wall with glass beads. Similar results have been reported by Dulski and Turner (1988) with 51% of recovered sporocysts. Mechanical grinding with glass beads is not likely to destroy every single oocyst’s wall. Excess of mechanical impact bears the risk of destruction of already liberated sporocysts and sporozoites. Alternatively, a mortar and pestle can also be used to grind oocysts instead of glass beads. Doran and Farr (1962) report a recovery of 30–65% of available *E. acervulina* sporocysts using the mortar method. Furthermore, in our new protocol, an average of only 3.33% of excysted sporozoites was lost during purification after sporocysts excystation. These results could be similar to those reported by Schmatz et al. (6 to 0% loss) if their recovery percentage was calculated from the total of excysted sporozoites. Dulski and Turner (1988) described only 1% loss of sporozoites after Percoll purification. However, that method comprises two Percoll gradient centrifugations of 20 min each which is time-consuming.

Research on chicken *Eimeria* spp. brings insights into coccidian metabolism, genetics, epidemiology and host-parasite interaction (Györke et al. 2013, Blake et al. 2015, Zhang et al. 2015). These developments could translate into improved therapies or preventive measurements. As ethically responsible study designs, in vitro models are pivotal to fulfil the goals of the 3R principle in animal research (Russell and Burch 1959). *Eimeria* sporozoites are commonly used in in vitro studies (Zhou et al. 2013, Thabet et al. 2015, Zhang et al. 2015, Bussière et al. 2018, Zhang et al. 2018). Sporozoites are the first cell invasive stage during eimeriosis. Therefore, any research on in vitro features of these coccidia depends on availability of viable sporozoites.

The development of a time saving and economic alternative to current methods for sporozoite purification could increase the interest in eimeriosis research. Furthermore, this protocol was established in *E. tenella*. Therefore, applications of this modified method in further *Eimeria* species should be encouraged and likewise reported.
